# Genome-Wide Association Study and Genomic Selection for Proteinogenic Methionine in Soybean Seeds

**DOI:** 10.3389/fpls.2022.859109

**Published:** 2022-04-25

**Authors:** William M. Singer, Zachary Shea, Dajun Yu, Haibo Huang, M. A. Rouf Mian, Chao Shang, Maria L. Rosso, Qijan J. Song, Bo Zhang

**Affiliations:** ^1^School of Plant and Environmental Sciences, Virginia Tech, Blacksburg, VA, United States; ^2^Department of Food Science and Technology, Virginia Tech, Blacksburg, VA, United States; ^3^Soybean and Nitrogen Fixation Unit, United States Department of Agriculture-Agricultural Research Service (USDA-ARS), Raleigh, NC, United States; ^4^Soybean Genomics and Improvement Laboratory, Beltsville Agricultural Research Center, United States Department of Agriculture-Agricultural Research Service (USDA-ARS), Beltsville, MD, United States

**Keywords:** soybean protein, soybean amino acid, methionine, sulfur-containing amino acid, GWAS, genomic selection

## Abstract

Soybean [*Glycine max (L.) Merr.*] seeds have an amino acid profile that provides excellent viability as a food and feed protein source. However, low concentrations of an essential amino acid, methionine, limit the nutritional utility of soybean protein. The objectives of this study were to identify genomic associations and evaluate the potential for genomic selection (GS) for methionine content in soybean seeds. We performed a genome-wide association study (GWAS) that utilized 311 soybean accessions from maturity groups IV and V grown in three locations in 2018 and 2019. A total of 35,570 single nucleotide polymorphisms (SNPs) were used to identify genomic associations with proteinogenic methionine content that was quantified by high-performance liquid chromatography (HPLC). Across four environments, 23 novel SNPs were identified as being associated with methionine content. The strongest associations were found on chromosomes 3 (ss715586112, ss715586120, ss715586126, ss715586203, and ss715586204), 8 (ss715599541 and ss715599547) and 16 (ss715625009). Several gene models were recognized within proximity to these SNPs, such as a leucine-rich repeat protein kinase and a serine/threonine protein kinase. Identification of these linked SNPs should help soybean breeders to improve protein quality in soybean seeds. GS was evaluated using k-fold cross validation within each environment with two SNP sets, the complete 35,570 set and a subset of 248 SNPs determined to be associated with methionine through GWAS. Average prediction accuracy (*r*^2^) was highest using the SNP subset ranging from 0.45 to 0.62, which was a significant improvement from the complete set accuracy that ranged from 0.03 to 0.27. This indicated that GS utilizing a significant subset of SNPs may be a viable tool for soybean breeders seeking to improve methionine content.

## Introduction

Soybean [*Glycine max (L.) Merr.*] has an ideal amino acid profile among the protein sources used in livestock feed and human food. All nine essential amino acids, histidine (His), isoleucine (Ile) leucine (Leu), lysine (Lys), methionine (Met), phenylalanine (Phe), threonine (Thr), tryptophan (Trp), and valine (Val), are present in soybean seeds (Kuiken and Lyman, 1949; Boisen et al., 2000). Accounting for 35% of the seed (Wilson, 2004), the protein component is processed into meal and regularly used in cattle, swine, and poultry feed (Buttery and D’Mello, 1994). During 2020, 33.2 million metric tons of soybean meal were used in the United States for livestock feed, in which 20.2, 6.3, and 5.8 million metric tons were fed to poultry, swine, and cattle, respectively (The American Soybean Association, 2020).

While all essential amino acids are present, soybean is deficient in Met which limits its nutritional utility in feed (Berry et al., 1962; Fernandez et al., 1994; Bonato et al., 2011). Met is required for metabolic processes and is the initiating amino acid in protein synthesis (Brosnan et al., 2007). Due to Met deficiency, poultry has displayed negative effects on body composition such as protein, fat, and tissue gain (Conde-Aguilera et al., 2013) and disease immunity (Wu, 2014). For this reason, synthetic supplementation of Met is critical to livestock feed, especially poultry. Bunchasak (2009) summarized the importance, viability, and special considerations for Met supplementation, however, synthetic methionine production generates hazardous waste and contributes to the greater dependence on fossil fuels (Willke, 2014; Neubauer and Landecker, 2021). Therefore, a sustainable solution would be increasing Met concentrations in soybean protein through breeding.

Since soybean was introduced to North America in 1765 (Hymowitz and Harlan, 1983), it has gained global prevalence. Contemporary soybean breeders have dedicated enormous effort to improve seed composition. Patil et al. (2017) aptly reviewed and described modern genomic efforts to improve soybean protein content. More specifically, quantitative trait loci (QTL) have been identified for protein concentration (Panthee et al., 2005; Warrington et al., 2015) as well as amino acid profiles (Panthee et al., 2006a,b; Fallen et al., 2013; Warrington et al., 2015; Li et al., 2018). Direct breeding results from this research include the sole publicly developed United States soybean variety (TN04–5321) release with enhanced sulfur-containing amino acids concentrations (Panthee and Pantalone, 2006) and potential introgression of an allele for significantly increased protein content (Warrington et al., 2015). Additionally, recent advances in molecular markers and high-throughput sequencing, summarized well by Zargar et al. (2015), have allowed for genomic research at the genome-wide level. Hwang et al. (2014) and Li et al. (2019) used single nucleotide polymorphisms (SNPs) to pinpoint genetic control of protein in soybean seed through genome-wide association studies (GWAS). Lee et al. (2019) targeted protein content as well as four amino acids, Met, Cys, Lys, and Thr, through GWAS. Qin et al. (2019) used GWAS to find genomic associations for 15 amino acids, Ala, Arg, Asp, Glu, Gly, His, Ile, Leu, Lys, Phe, Pro, Ser, Thr, Tyr, and Val. A single study also focused directly on Met and Cys with genome-wide associations for Canadian soybean lines in MG 000-II (Malle et al., 2020). Lee et al. (2019) and Malle et al. (2020) reported Met measurements using near-infrared reflectance spectroscopy (NIRS), whereas Qin et al. (2019) utilized ion-exchange chromatography.

Genomic selection (GS) utilizes similar statistical models as GWAS, but it seeks to exploit larger genomic variations than individual genomic regions (Meuwissen et al., 2001). GS has been shown to reduce selection time in soybean breeding (Matei et al., 2018) and the United States soybean germplasm collection has proven to be a valuable resource for creating GS models (Jarquin et al., 2016). Promising results have displayed successful prediction of grain yield, protein and oil content, plant height, maturity, seed weight (Ma et al., 2016; Duhnen et al., 2017; Stewart-Brown et al., 2019; Ravelombola et al., 2021) as well as soybean cyst nematode resistance (Ravelombola et al., 2019, 2020). However, only one study by Qin et al. (2019) has evaluated GS for amino acid content in soybean seed, and it did not include Met concentrations.

Additionally, Warrington et al. (2015) identified negative correlations between increased protein content and Lys, Thr, and Met+Cys concentrations. This suggests complex genetic controls of protein as soybean breeders balance objectives for protein quantity and quality moving forward. Therefore, this project seeks to further elucidate genomic associations through GWAS and evaluate the potential for GS of proteinogenic Met content in soybean seeds.

## Materials and Methods

### Plant Materials

A total of 500 soybean accessions were selected from the USDA Soybean Germplasm Collection to represent maximum genetic variability in maturity groups IV and V based on genetic distance (Qin et al., 2017). Among them, a panel consisting of 311 accessions from 17 different countries (Table 1) with good seed quality, i.e., without discoloration, mottling, and visible disease, were grown in 3 m two-row plots with 76 cm row spacing in Blacksburg, VA and 4.2 m single row plots with 96 cm row spacing in Clayton, NC in 2018. They were also grown in 3 m four-row plots with 76 cm row spacing in Warsaw, VA and repeated in Blacksburg, VA. Plots were organized based upon maturity and grown as a randomized complete block design (RCBD) with two blocks at each location. Each block included two commercial checks, Ellis and AG4403. Due to limited seed quantity in general, block replicates were merged prior to seed processing.

**TABLE 1:** Countries of origin and maturity groups (MG) for clustered accessions as determined by discriminant analysis of principal components (DAPC).

	Cluster 1		Cluster 2		Cluster 3		Cluster 4		
	(*n* = 76)		(*n* = 62)		(*n* = 47)		(*n* = 126)		
	Count	%	Count	%	Count	%	Count	%	Total
Australia	–	–	–	–	1	2.1	–		1
Brazil	–	–	–	–	1	2.1	1	0.8	2
China	55	72.4	54	87.1	–	–	65	51.6	174
Costa Rica	–	–	–	–	1	2.1	–		1
Georgia	–	–	1	1.6	–	–	2	1.6	3
India	–	–	–	–	–	–	1	0.8	1
Indonesia	1	1.3	–	–	–	–	–		1
Japan	5	6.6	4	6.5	2	4.3	15	11.9	26
Morocco	–	–	–	–	–	–	1	0.8	1
Nepal	–	–	–	–	–	–	1	0.8	1
North Korea	–	–	–	–	–	–	7	5.6	7
Russia	–	–	–	–	–	–	1	0.8	1
South Korea	–	–	1	1.6	3	6.4	14	11	18
Taiwan	3	3.9	–	–	–	–	1	0.8	4
Uganda	–	–	–	–	–	–	2	1.6	2
United States	–	–	2	3.2	37	78.7	11	8.7	50
Vietnam	11	14.5	–	–	–	–	2	1.6	13
Unknown	1	1.3	–	–	2	4.3	2	1.6	5
MG IV	36	47.4	52	83.9	37	78.7	97	77	222
MG V	40	52.6	10	16.1	10	21.3	29	23	89

### Data Collection

All seed samples were cleaned by removing moldy, mottled, discolored, or off-types seeds. Dry-matter based protein content and moisture were measured using the DA 7250 NIR Analyzer spectrophotometer (PerkinElmer Inc.) through near-infrared reflectance spectroscopy (NIRS). For NIRS, the manufacturer’s annual updated calibration module was used and protein content was recorded for each sample.

Samples were ground using a water-cooler Foss 1095 Knifetec mill to a consistent particle size. Subsamples of 0.01 g were weighed into glass digestion tubes and subsequently hydrolyzed using a modified method 994.12 (Aoac International, 2021) to break apart proteinogenic methionine. Samples were first oxidized with 0.5 mL of performic acid at 0°C for 16 h and 200 μL of sodium metabisulfite solution was added to end the reaction. Hydrolysis was then performed with 3 mL of 6 M HCl at 110°C for 16 h. Next, samples were diluted to 10 mL with water, and 750 μL subsamples were taken and centrifuged under vacuum to remove HCl.

Concentrated samples were rehydrated with water into vials for high-performance liquid chromatography (HPLC) analysis. HPLC was performed using online derivatization with o-phthalaldehyde (OPA), ultra-violet (UV) detection, and the Agilent AdvanceBio Amino Acid Analysis (AAA) 4.6 × 100 mm, 2.7 μm LC column and 4.6 × 5 mm guard columns with the Agilent HPLC model 1200. Each sample had two technical replicates that were averaged to account for biological and equipment variation. To better describe proteinogenic concentrations, Met was reported on a g/kg crude protein (g kg^–1^ cp) basis. Data were fit with an ANOVA using standard least squares that included accession, location, and year as fixed effects.

### Genotypic Data

Publicly available SNP marker data^1^ of the 311 accessions were downloaded from the SoySNP50K SNPs data repository (Song et al., 2015). A total of 42,509 initial SNPs were filtered by low minor allele frequency (MAF < 0.05) and missing genotypes, which resulted in 35,570 SNPs being used for further analysis.

### Population Structure

Population structure was evaluated through a discriminant analysis of principal components (DAPC) using the *adegenet* package (Jombart, 2008) in R to identify clusters of genetically related individuals (Jombart et al., 2010). Successive k-means clustering with the function *find.clusters* with maximum clusters as *k* = 40 was used. A total of 300 principal components were retained, and Bayesian information criterion (BIC) was used to identify an optimal number of clusters. The function *dapc* was then used by retaining an optimal number of principal components to maximize cumulative variance without overfitting, and all discriminant functions and eigenvalues were retained. A kinship matrix was also created with the software TASSEL 5 (Bradbury et al., 2007) using the Centered_IBS method (Endelman and Jannink, 2012).

### Genome-Wide Association Analysis and Candidate Gene Evaluation

Associations between genotypic and phenotypic data were analyzed using two different models in TASSEL 5: mixed linear model (MLM) and general linear model (GLM). Predominantly, MLM was used to incorporate a kinship matrix (K) jointly with population structure (Q) for increased statistical power through the Q+K approach (Yu et al., 2006). GLM was used to examine individual location datasets through a more lenient least squares fixed effect model with Q as a covariate. Additionally, five principal components (accounting for 18.75% cumulative variance) were included as covariates for the 2018 Blacksburg, VA and 2019 Warsaw, VA datasets to better control for false positive associations. A modified Šidák correction (α_*sid*_ = 1−(1−α)^(1/*m*)^) for multiple testing was used to identify significant associations. The effective number of markers (*M*_*eff*_) was calculated to be 4,191 using the *poolr* package in R with the Li and Ji method (Li and Ji, 2005). *M*_*eff*_ replaced *m*, and thus, the adjusted significance threshold at α = 5% and the suggestive threshold at α = 25% were −log_10_(*P*) > 4.91 and −log_10_(*P*) > 4.16, respectively. QQ and Manhattan plots were used to visualize results with the *qqman* package (Turner, 2014). Gene models from Glyma.Wm82.a2.v1 (Williams 82) as displayed on^2^ within 10 kb of significant SNPs flanking regions were reported as candidate genes (Xie et al., 2018; Qin et al., 2019). Gene descriptions were reported from gene homolog descriptions from TAIR for *Arabidopsis thaliana* (Berardini et al., 2015). If TAIR homologs were not available, descriptions were reported from either PANTHER or GO databases (Ashburner et al., 2000; Mi et al., 2013; Gene Ontology Consortium, 2021). Expression patterns within soybean reproductive tissues (flowers, pods, and seeds) of each gene model were also reported when available (Severin et al., 2010).

### Genomic Selection

Genomic selection was performed using gBLUP (genomic best linear unbiased prediction) with the TASSEL 5 genomic selection function. Similar to the GWAS, the Q+K approach was used to fit a mixed model with population structure and a kinship matrix as covariates. K-fold cross validation was performed using *k* = 5 with 20 iterations, and the coefficient of determination (*r*^2^) was collected for each fold. Each environment’s dataset underwent GS using all 35,570 SNPs as well as a subset of 248 SNPs generated with a significance threshold of −log_10_(*P*) > 3 from the GWAS (Qin et al., 2019). A *T*-test was used to compare *r*^2^ values between the whole and partial SNP models.

## Results

### Phenotype

**FIGURE 1:**
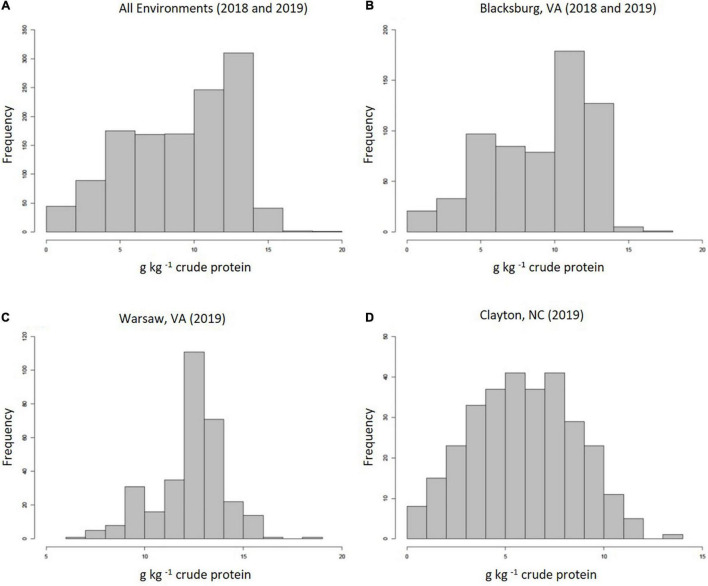
Frequency distributions displaying proteinogenic Met concentrations collected from all environments **(A)**, Blacksburg, VA **(B)**, Warsaw, VA **(C)**, and Clayton, NC **(D)**.

Methionine concentrations across all environments displayed normal, continuous distributions with a grand mean of 9.06 g kg^–1^ cp and an average standard deviation (SD) of 2.84 g kg^–1^ cp. Figure 1 highlights distributions for all environments combined (1a), 2018 and 2019 Blacksburg, VA (1b), Warsaw, VA (1c), and Clayton, NC (1d). Blacksburg, Warsaw, and Clayton environments had means and SDs of 8.96, 12.32, and 5.88 g kg^–1^ cp and 3.36, 1.73, and 2.61 g kg^–1^ cp, respectively. Warsaw, VA exhibited significantly higher average Met than both other locations, while Blacksburg, VA also possessed significantly higher average Met than Clayton, NC. Samples grown in 2019 showed significantly higher Met content than 2018, but accessions were not shown to have a significant impact on Met content.

### Population Structure

**FIGURE 2:**
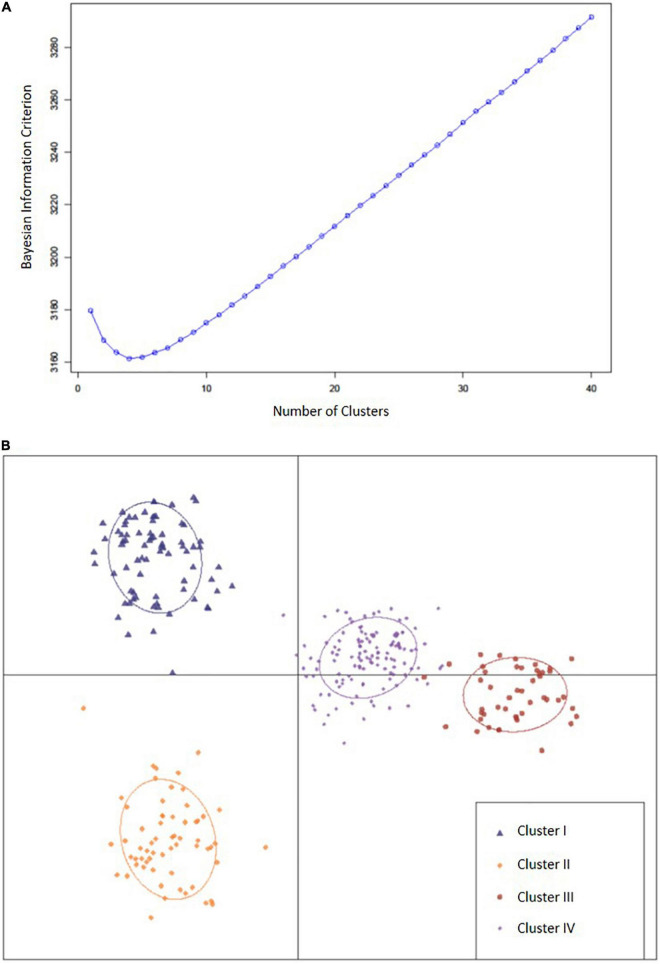
**(A)** Bayesian information criterion for selecting the optimal number of clusters. **(B)** A scatter plot depicting the four clusters (*k* = 4) identified as likely subpopulations within the 311 accessions: cluster I (blue triangle, *n* = 76), cluster II (gold diamonds, *n* = 62), cluster III (large red circles, *n* = 47), cluster IV (small purple circles, *n* = 126).

Through DAPC, 150 principal components that accounted for 78% of cumulative variance were retained, and with the smallest BIC, *k* = 4 was determined as the optimal number of clusters (Figure 2). Country of origin for accessions within each cluster were identified (Table 1). Cluster I (*n* = 76) contained 55 accessions (72.4%) that originated from China, 11 from Vietnam (14.5%), five from Japan (6.6%), three from Taiwan (3.9%), and one from Indonesia (1.3%). Cluster I also contained 52.6% of accessions from maturity group (MG) V. Cluster II (*n* = 62) contained 54 (87.1%), four (6.5%), two (3.2%), one (1.6%), and one (1.6%) accessions from China, Japan, the United States, Georgia, and South Korea, respectively, and 83.9% of those belonged to MG IV. Cluster III (*n* = 47) contained 37 (78.7%) accessions from the United States, three (6.4%) from South Korea, two (4.3%) from Japan, and one (2.1% each) from Australia, Brazil, and Costa Rica. Cluster III also contained 78.7% of accessions from MG IV. Cluster IV (*n* = 126) contained 65 (51.6%), 15 (11.9%), 14 (11%), 11 (8.7%), and seven (5.6%) accessions from China, Japan, South Korea, the United States, and North Korea, respectively, as well as two (1.6% each) accessions from Georgia, Uganda, and Vietnam and one accession (0.8% each) from Brazil, India, Morocco, Nepal, Russia, and Taiwan. Within cluster IV, 77% of accessions belonged to MG IV. Clusters were not shown to have a significant effect on Met content. Although, the clusters displayed that accession were stratified predominantly by geographic origin which proved useful in identifying genetically similar accessions.

### Genome-Wide Associations

**FIGURE 3:**
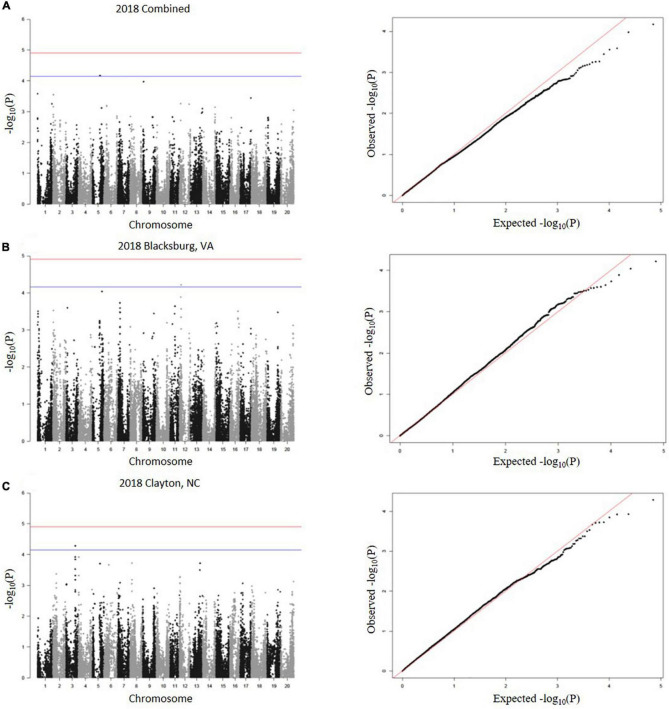
SNP associations for 2018 environments, **(A)** combined, **(B)** Blacksburg, VA, **(C)** Clayton, NC, are displayed in Manhattan plots with chromosomes in alternating colors, significance thresholds-log_10_(P) > 4.91 and suggestive threshold-log_10_(P) > 4.16. Each respective QQ plot displays observed-log_10_(P) against expected-log_10_(P).

**FIGURE 4:**
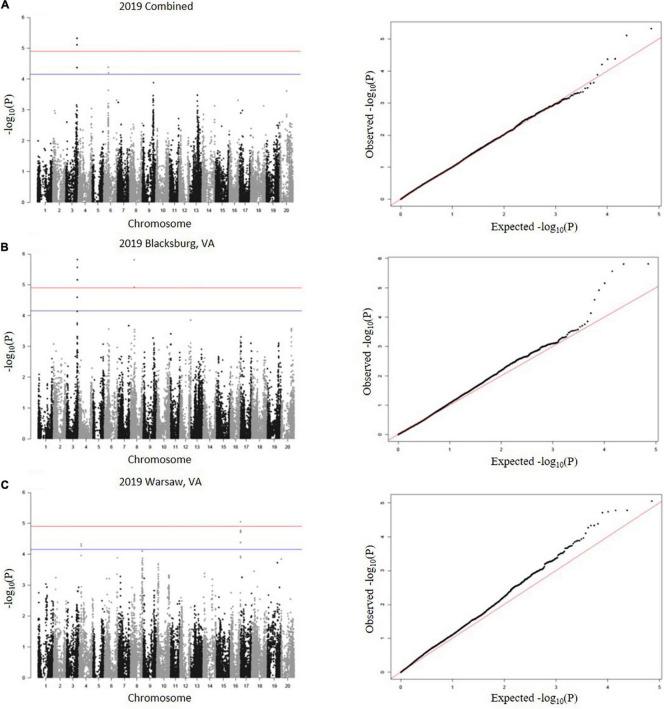
SNP associations for 2019 environments, **(A)** combined, **(B)** Blacksburg, VA, **(C)** Warsaw, VA are displayed in Manhattan plots with chromosomes in alternating colors, significance threshold-log_10_(P) > 4.91 and suggestive threshold-log_10_(P) > 4.16. Each respective QQ plot displays observed-log_10_(P) against expected-log_10_(P).

A total of 23 SNPs were identified as being associated with proteinogenic Met concentration (g kg^–1^ cp) in soybean seed (Table 2). MLM and GLM models from 2018 environments displayed three SNPs (one SNP from each model) above the suggestive threshold (Figure 3), whereas MLM and GLM models from 2019 environments displayed 20 SNPs above the suggestive threshold (six from Blacksburg, VA, nine from Warsaw, VA, and five from a combined locations) (Figure 4). QQ plots for each model exhibited that Type I and Type II errors were accounted for sufficiently (Figures 3, 4). Eight SNPs displayed significant associations [−log_10_(P) > 4.91]: ss715586112, ss715586120, ss715586126, ss715586203, ss71558 6204, ss715599541, ss715599547, and ss715625009. The remaining 15 SNPs displayed −log_10_(P) > 4.16 which was above the suggestive threshold: ss715585365, ss715586063, ss715 586201, ss715589347, ss715589348, ss715589349, ss715590327, ss715593682, ss715593752, ss715625002, ss715625007, ss715625012, ss715625013, and ss715625017. Chromosome (Chr) 3 contained the most associations (five significant, three suggestive), followed by Chr 16 (one significant, five suggestive), Chr 4 (three suggestive), Chr 6 (two suggestive), Chr 8 (two significant), Chr 5 (one suggestive), and Chr 12 (one suggestive). When including all environments, an MLM did not identify any SNPs above the significance or suggestive threshold.

**TABLE 2:** Significant SNPs on chromosomes 3, 4, 5, 6, 8, 12, and 16 associated with Met content (g kg^–1^ cp) in soybean seeds.

Chr	Genomic location	SNP (position)	Wm82 Allele^a^	Alter-native Allele	Environments^d^					
					2018 BB	2018 CL	2018 Combined	2019 BB	2019 W	2019 Combined

					——————————————— -log_10_(P) —————————————–					
3	Intergenic	ss715585365 (33765404)	T	G	NS^b^	4.29*	NS	NS	NS	NS
	Intergenic	ss715586063 (39357229)	C	T	NS	NS	NS	4.60*	NS	NS
	Intergenic	ss715586112 (39946374)	A	G	NS	NS	NS	5.82**	NS	NS
	Intergenic	ss715586120 (40006278)	A	G	NS	NS	NS	5.16**	NS	NS
	Coding sequence	ss715586126 (40062294)	T	G	NS	NS	NS	5.57**	NS	NS
	Intergenic	ss715586201 (41217558)	A	G	NS	NS	NS	NS	NS	4.37*
	Coding sequence	ss715586203 (41228895)	G	T	NS	NS	NS	NS	NS	5.33**
	Intergenic	ss715586204 (41236923)	G	A	NS	NS	NS	NS	NS	5.11**
4	Coding sequence	ss715589347 (8089953)	T	C	NS	NS	NS	NS	4.27*	NS
	Intron	ss715589348 (8091107)	G	A	NS	NS	NS	NS	4.33*	NS
	Coding sequence	ss715589349 (8095691)	C	T	NS	NS	NS	NS	4.33*	NS
5	Intergenic	ss715590327 (27762168)	A	G	NS	NS	4.17*	NS	NS	NS
6	Coding sequence	ss715593682 (17154269)	G	A	NS	NS	NS	NS	NS	4.39*
	Intergenic	ss715593752 (17453327)	C	T	NS	NS	NS	NS	NS	4.20*
8	3′ UTR^c^	ss715599541 (14196322)	T	C	NS	NS	NS	4.92**	NS	NS
	Intergenic	ss715599547 (14226774)	G	A	NS	NS	NS	5.81**	NS	NS
12	Intergenic	ss715613175 (5433032)	T	G	4.22*	NS	NS	NS	NS	NS
16	Intron	ss715625002 (37660795)	A	C	NS	NS	NS	NS	4.78*	NS
	Intergenic	ss715625007 (37701598)	T	G	NS	NS	NS	NS	4.38*	NS
	Intergenic	ss715625009 (37712387)	T	C	NS	NS	NS	NS	5.05**	NS
	Coding sequence	ss715625012 (37737235)	C	T	NS	NS	NS	NS	4.71*	NS
	Intergenic	ss715625013 (37753573)	T	C	NS	NS	NS	NS	4.74*	NS
	Intergenic	ss715625017 (37784014)	T	C	NS	NS	NS	NS	4.78*	NS

** significance threshold (5%), * suggestive threshold (25%). ^a^Williams 82. ^b^not significant. ^c^3 prime untranslated region. ^d^Blacksburg, VA (BB), Clayton, NC (CL), Warsaw, VA (W).

### Candidate Genes

A total of 22 candidate gene models from Wm82 were found within 10 kb flanking regions of each significant SNP (Table 3). A number of gene models were found on three chromosomes: 13 on Chr 3 (Glyma.03g188100, Glyma.03g18 8200, Glyma.03g188300, Glyma.03g188400, Glyma.03g188900, Glyma.03g189000, Glyma.03g189100, Glyma.03g189700, Glyma. 03g189800, Glyma.03g203900, Glyma.03g204000, Glyma.03g20 4100, and Glyma.03g204200), seven on Chr 8 (Glyma.08g177000, Glyma.08g177100, Glyma.08g177200, Glyma.08g177300, Glyma.08g177400, Glyma.08g177500, and Glyma.08g177600), and two on Chr 16 (Glyma.16g219800 and Glyma.16g219900). Candidate gene models belong to several protein families with numerous metabolic and biosynthesis implications. Of the 13 genes present on Chr 3, nine displayed moderate to high expression in reproductive tissues. Specifically, Glyma.03g188900, a ubiquitin-protein ligase, and Glyma.03g189800, a leucine-rich repeat (LRR) protein kinase, displayed high expression in all reproductive tissue and pods, respectively. On Chr 8, four out of seven genes had moderate to high expression in reproductive tissue, including Glyma.08g177000 a RING/U-box superfamily protein. On Chr 16, Glyma.16g219800 displayed little to no expression in reproductive tissue, and Glyma.16g219900 did not have available expression data.

**TABLE 3:** Candidate gene models and descriptions within 10 kb flanking regions of significantly associated SNPs using Wm82.a2.v1.

Chr	SNP	Candidate genes	Gene function description^a^	Expression in soybean reproductive tissue^b^
3	ss715586112	Glyma.03g188100	Modifier of rudimentary protein	High expression in flowers
		Glyma.03g188200	Nucleic acid binding	NA
		Glyma.03g188300	Pollen Ole e 1 allergen and extensin family protein	Little to no expression in reproductive tissue
		Glyma.03g188400	Eukaryotic aspartyl protease family protein	Moderate to high expression in seeds and pods
	ss715586120	Glyma.03g188900	Ubiquitin-protein ligase 7	High expression in flowers, pods, and seeds
		Glyma.03g189000	Pentatricopeptide repeat (PPR) superfamily protein	Moderate to high expression in flowers, pods, and seeds
		Glyma.03g189100	Exostosin family protein	Moderate to high expression in seeds
	ss715586126	Glyma.03g189700	Pyruvate kinase family protein	Moderate to high expression in seeds
		Glyma.03g189800	Leucine-rich repeat (LRR) protein kinase family protein	High expression in pods
	ss715586203	Glyma.03g203900	Polyketide cyclase/dehydrase/lipid transport superfamily protein	NA
		Glyma.03g204000	Mal d 1-associated protein	Moderate expression in flowers, pods, and seeds
		Glyma.03g204100	Calmodulin-domain protein kinase cdpk isoform 2	Moderate to high expression in pods
	ss715586204	Glyma.03g204200	TPX2 (targeting protein for Xklp2) protein family	Little to no expression in reproductive tissue
8	ss715599541	Glyma.08g177000	RING/U-box superfamily protein	High expression in flower and pods
		Glyma.08g177100	NA	Little to no expression in reproductive tissue
		Glyma.08g177200	Arabinogalactan protein 1	NA
		Glyma.08g177300	GTP cyclohydrolase II	Little to no expression in reproductive tissue
	ss715599547	Glyma.08g177400	Dicarboxylate transport 2.1	Moderate expression in pods and seeds
		Glyma.08g177500	Pyrimidine 2	Moderate expression in flowers
		Glyma.08g177600	Centrin2	High expression in flowers; moderate expression in pods
16	ss715625009	Glyma.16g219800	WRKY DNA-binding protein 70	Little to no expression in reproductive tissue
		Glyma.16g219900	B-block binding subunit of TFIIIC	NA

*^a^as described in TAIR, PANTHER, or GO annotation.*

*^b^Soybean flowers, seeds, and pods. Detailed expression profiles can be found in Severin et al. (2010).*

### Genomic Selection

**FIGURE 5:**
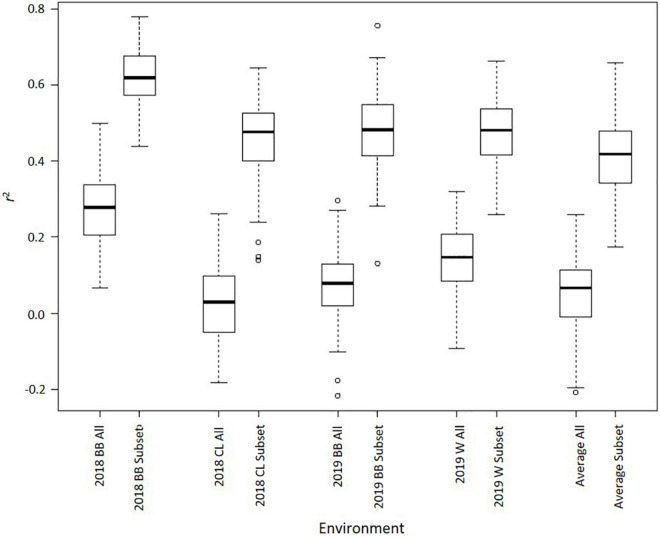
Boxplots displaying 100 r^2^ values (*k* = 5, 20 iterations) for GS models using 35,570 SNPs (ALL) and 248 SNPs (Subset) across environments (BB = Blacksburg, VA; CL = Clayton, NC; W = Warsaw, VA; Average = Mean met across all environments).

Genomic best linear unbiased prediction through TASSEL estimated GEBVs using two different sets of SNPs: a complete set with 35,570 SNPs and a subset of 248 SNPs with some association [−log_10_(*P*) > 3] with Met content. The 248 SNP subset is displayed in Supplementary Material 1. The coefficient of determination (*r*^2^) between GEBVs and observed values varied throughout environments, but the subset of 248 SNPs consistently outperformed the larger SNP set (Figure 5). Using the larger set, the average *r*^2^ for 2018 Blacksburg, VA, 2018 Clayton, NC, 2019 Blacksburg, VA, and 2019 Warsaw, VA datasets was 0.27, 0.03, 0.08, and 0.14, respectively. Using the 248 SNP subset, the average *r*^2^ for 2018 Blacksburg, VA, 2018 Clayton, NC, 2019 Blacksburg, VA, and 2019 Warsaw, VA datasets was 0.62, 0.45, 0.48, and 0.48, respectively. When averaging Met content across all environments, prediction accuracy remained consistent, 0.05 and 0.41 average *r*^2^ for the complete set and subset, respectively. *T*-tests comparing *r*^2^ between SNP sets within environments identified that accuracy when using the subset was significantly higher across all environments (*P* < 0.01).

## Discussion

Soybean protein content and amino acid profiles are critical objectives for plant breeders. For this reason, many resources have been allocated to unlock genomic controls for these traits. As suggested by Jarquin et al. (2016) and Lee et al. (2019), utilizing the high-density marker set from the SoySNP50K repository with environmentally suitable accessions in replicated, multi-location trials is a powerful method for revealing genetic potential. In this study, we identified novel associations for proteinogenic Met content (g kg^–1^ cp) in soybean seeds using accessions from MG IV and V that complements current genomic knowledge. Furthermore, we discovered that GS with a subset of significantly associated SNPs improved the genomic prediction accuracy for Met.

Previous studies have identified genomic associations with Met content on chromosomes 1, 2, 6, 7, 9, 10, 11, 12, 13, 14, 15, 17, 18, and 20 (Panthee et al., 2006a; Fallen et al., 2013; Kastoori et al., 2014; Warrington et al., 2015; Zhang et al., 2018; Lee et al., 2019; Malle et al., 2020). Although our study did not identify these same genetic regions, ss715593752 on Chr 6 was within 220 kb of a QTL from Warrington et al. (2015) and a suggested SNP from Lee et al. (2019). Additionally, ss715593682 is within 6,000 kb of a SNP identified by Zhang et al. (2018). Through GWAS, we identified 23 novel SNP associations for proteinogenic Met content that were not recurrent across environment, which is consistent with previous research (McClure et al., 2017; Lee et al., 2019). This suggests further research is needed to understand GxE interactions for amino acid profile improvements in soybean due to their complexity.

Our analyses identified associations greater in number and significance from the 2019 dataset when compared to 2018 measurements. This is likely caused by substantial differences between Met concentrations between environments including soil type and rainfall. Environment temperature was also considered, but there was little to no difference between locations besides slightly lower temperatures in Blacksburg, VA as a function of elevation. As shown in Figure 1, the histogram for Warsaw, VA displays an expected frequency distribution for Met content, whereas other distributions exhibit numerous measurements below expected levels as a result of included 2018 data. Soil type varied in each environment with loamy sand being present in Clayton, NC and different combinations of loam and silt loam, and loam being present in Blacksburg, VA, and Warsaw, VA (Soil Survey Staff, 2022). Furthermore soybeans harvested from both locations in 2018 exhibited poorer seed quality likely as a function of higher than normal precipitation rates late in the growing season and delayed harvest. Rainfall, specifically in September and October, was significantly higher during 2018. When comparing Blacksburg, VA environments, rainfall was 10 cm higher in 2018, and rainfall in Clayton, NC was 14 cm higher than 2019 Blacksburg, VA and 18 cm higher than Warsaw, VA. Rainfall has been shown to have a negative correlation with protein content (Kumar et al., 2006) and delayed harvest dates decrease concentrations of seed components (Jaureguy et al., 2013). These factors combined with higher disease rates, due to increased moisture, likely had negative impacts the proteinogenic Met content. Overall, Clayton, NC had the most environment discrepancies with higher sand percentages in soil and rainfall amounts while 2018 Blacksburg, VA also suffered from high rainfall and delayed harvest.

The three SNP associations from 2018 data exhibited a -log_10_(P) greater than the suggestive threshold, but not the significance threshold. Although, ss715590327 (suggested from combined 2018 environments) was within 10 kb of Glyma.05g104400, a gene model involved in peptidyl-amino acid modification. The 20 SNPs identified from our 2019 datasets provide superior evidence for associations to Met concentrations. The strongest associations occurred on Chr 3 with a set of four SNPs (ss715586063, ss715586112, ss715586120, and ss715586126) within a distance of 710 kb and another set of three SNPs (ss715586201, ss715586203, and ss715586204) within a distance of 20 kb. Within immediate proximity to the former set, nine gene models of relevant protein functions are present with ss715586126 being inside the coding region of Glyma.03g18980, a leucine-rich repeat protein kinase family protein that is highly expressed in pod walls. The latter set is close to four gene models including Glyma.03g204000, a Mal d 1-associated protein expressed highly in the root system and moderately in pods and developing seeds, where ss715586203 is within the coding sequence.

While only suggestive associations, two SNPs on Chr 6 are within a 300 kb distance, and ss715593682 is part of the coding region for a S-adenosyl-L-methionine-dependent methyltransferase, Glyma.06g193300. The two significant SNPs found on Chr 8 (ss715599541 and ss715599547) are within 31 kb of each other and are proximal to seven various genes. Interestingly, ss715599541 is a part of the 3’ untranslated region of Glyma.08g177100, a gene model with unknown function. Chr 16 contains one significant SNP association (ss715625009) that is flanked by five other suggestive associations, all within a 124 kb region. Within this region, ss715625012 can be found in the coding sequence of Glyma.16g220200, a serine/threonine protein kinase.

When our results are combined with previously identified marker-trait associations, genomic regions impacting Met concentration in soybean seeds can be found on all chromosomes except Chr 19. This creates a complicated framework for increasing Met content through marker-assisted selection (MAS), transgenic, or genome editing approaches. Amir et al. (2019) summarized current efforts at biofortification of Met in plant seeds through gene regulation and found that most attempts failed to increase Met in a synergistic manner. More specifically, some researchers have incorporated cystathionine γ-synthase genes from *Arabidopsis thaliana* into soybean; Song et al. (2013) found an increase in general Met content, whereas Hanafy et al. (2013) saw increased soluble Met but not total Met in seeds. In *Arabidopsis thaliana*, Girija et al. (2020) discovered that Met protein residues, unsoluble Met production was the limiting factor for final Met content in seeds.

In breeding applications, our study suggests that GS may be a useful tool for selecting varieties with increased Met content. GS success is mainly determined by prediction accuracy (Duhnen et al., 2017) and impacted by many variables, including marker density. While high-density marker sets are typically ideal for utilizing genome-wide data, subsets of significant SNPs have been found to perform equal to or better than large SNP collections (Zhang et al., 2016; Qin et al., 2019). Qin et al. (2019) specifically identified improved genomic prediction for soybean amino acid content using a subset of 231 SNPs. Our results showed similar improvement in prediction accuracies with a subset of 248 SNPs. In 2018 Clayton, NC, both 2019 environments, and using average Met content, GS had average accuracy values between 0.41 and 0.48. This could prove useful to breeders and may complement the use of significant SNPs from the 2019 dataset with MAS. However, when using the 2018 Blacksburg, VA dataset, predictive accuracy reached an average of 0.62. Considering the single suggestive SNP identified through GWAS for this location, GS appears to provide greater utility.

In summary, this project included a GWAS that not only identified many SNPs associated with Met content but also characterized several genomic regions that appear relevant. Within these regions, numerous gene models are present and their expression may correlate to the desired trait. GS was also evaluated as a potential method for selecting soybean lines with higher Met content. GS appears to be useful in certain environments with a subset of SNPs and could complement or outperform MAS. However, GxE limitations are still present and may impact which genes are influencing the final Met concentrations. This will require further research to elucidate genomic control of Met concentrations in soybean seed.

## Data Availability Statement

The raw data supporting the conclusions of this article will be made available by the authors, without undue reservation.

## Author Contributions

WS collected the data, performed the analyses, and wrote the manuscript. QS assisted in germplasm selection. ZS assisted in germplasm population maintenance. DY, HH, CS, and MR assisted in designing phenotypic quantification. BZ and MM provided study formulation and analysis expertise. All authors edited and reviewed the manuscript.

## Conflict of Interest

The authors declare that the research was conducted in the absence of any commercial or financial relationships that could be construed as a potential conflict of interest.

## Publisher’s Note

All claims expressed in this article are solely those of the authors and do not necessarily represent those of their affiliated organizations, or those of the publisher, the editors and the reviewers. Any product that may be evaluated in this article, or claim that may be made by its manufacturer, is not guaranteed or endorsed by the publisher.

## Footnotes

1
www.soybase.org

2
www.soybase.org
